# In Vitro Screening Potential Antibacterial Properties of the Greek Oregano Honey against Clinical Isolates of *Helicobacter pylori*

**DOI:** 10.3390/foods10071568

**Published:** 2021-07-06

**Authors:** Chrysoula (Chrysa) Voidarou, Georgios Rozos, Athanasios Alexopoulos, Stavros Plessas, Ioanna Mantzourani, Elisavet Stavropoulou, Athina Tzora, Eugenia Bezirtzoglou

**Affiliations:** 1 Department of Agricultural Sciences, Faculty of Agricultural Sciences, University of Ioannina, 47100 Arta, Greece; clevervet@hotmail.com (G.R.); tzora@uoi.gr (A.T.); 2 Laboratory of Microbiology, Biotechnology & Hygiene, Department of Agricultural Development, Democritus University of Thrace, 68200 Orestiada, Greece; alexopo@agro.duth.gr (A.A.); splessas@agro.duth.gr (S.P.); imantzou@agro.duth.gr (I.M.); 3 Service de Medecine Interne et Service des Maladies Infectieuses, Centre Hospitalier Universitaire Valois (CHUV), Rue de Bugnon, 1011 Lausanne, Switzerland; elisabeth.stavropoulou@gmail.com; 4 Laboratory of Hygiene and Environmental Protection, Medical School, Democritus University of Thrace, 67100 Alexandroupolis, Greece; empezirt@med.duth.gr

**Keywords:** antibacterial, *Helicobacter pylori*, honey, oregano, urease

## Abstract

Oregano honey is an exceedingly rare and distinct product, not commercially available, produced by bees bred in oregano fields of alpine altitudes at the mountainous area of Epirus, Greece. In ethnic popular medicine, this product is used as a therapeutic in various gastric diseases. To test this hypothesis, 14 strains of *Helicobacter pylori* (*H. pylori*), 6 isolated from gastric ulcers and 8 from cases of clinical gastritis, were employed in the present study. The above bacterial strains were exposed to various concentrations (75% *v*/*v*, 50% *v*/*v*, 25% *v*/*v*, 12.5% *v*/*v*, and 6% *v*/*v*) of 50 oregano honey samples by using the agar well method and the inhibition zones observed around each well were recorded. Although the inhibitory zones of the *H. pylori* isolated from the gastric ulcers were wide enough (0–34 mm), those strains, in general, appeared more resistant than the other eight (0–58 mm). The same result was observed when the same strains were tested against six antibiotics used in clinical practice. Extracts of oregano honey were prepared by extraction with four different organic solvents. *N*-hexane and chloroform extracts had the most potent antibacterial action. Finally, pure oregano honey and diethyl ether extracts of honey showed significant inhibitory activity against urease secreted by the pathogen. These results strongly indicate the susceptibility of *H. pylori* strains to the oregano honey by more than one mode of action. Consequently, this variety of honey seems to have potential therapeutic properties against gastric ulcers and gastritis, thus explaining the preference of the locals towards this traditional remedy.

## 1. Introduction

Honey has been used as a common remedy in human medicine all over the world. Ancient and modern cultures recognize empirically the antimicrobial properties of honey [1]. Its use is recorded for 4000 years and today its therapeutic use is expanded and popular to such an extent that the term apitherapy has been coined to describe a large variety of therapeutic remedies originating from honey and other bee products [1,2,3]. Most scientific research studies focus on the healing properties of honey in various skin conditions [4,5,6], while only a few of these investigate the use of honey as a systemic medicine administrated *per os* [7].

Wild oregano honey is derived from the nectar collected by bees foraging in wild mountain oregano flowers in the mountainous area of Epirus, Greece. Unlike other bee-keeping practices that produce poorer kinds of honey, the bees involved in the production of wild honey are never fed sugar, and thus, the final product is completely made of natural origin and unprocessed. Moreover, the oregano honey is a natural source of essential trace elements and minerals such as Mg and K as well as of vitamin B complex, with intact enzymes since it is raw and unprocessed. Unlike most types of commercial honey, it is not heated, and therefore, its ingredients retain their biodrastic effect [8,9]. The bees are visiting wild plants and tree blossoms, including wild mountain oregano fields, thus concentrating their essence and various phytochemicals into the honey. It is these plants’ essence and their phytochemicals that provide this variety of honey a bitter flavor and potential beneficial properties. Thus, oregano honey combines several benefits associated with the powerful wild oregano pollution-free extracts and its medicinal wild plant essences [10,11,12]. As mentioned above, it is a source of potassium (0.7 g per 100 g), an important source of phosphorus, calcium, magnesium, natural enzymes, and phenolic acids [13]. The Greek oregano honey has a golden imposing color and a strong but not sweet flavor. It is a type of bitter honey without the expected typical sweet taste of other more common types of honey. Pure wild oregano honey is not easy to obtain because it is produced in small quantities in the alpine zone of the Epirus region in Greece. In the Greek popular therapeutic tradition, oregano honey is considered an excellent remedy for various gastric disorders and diseases, and many people suffering from various gastric ailments have tried it as an alternative more natural therapeutic approach (according to personal testimony by local people). These testimonies along with the powerful popular belief raise the question of whether oregano honey can be used as a “natural” medicine for the control of microbial infections, especially in the gastrointestinal tract [14,15,16]. In ailments of the gastrointestinal tract such as dyspepsia, stomach ulcer, gastritis, duodenitis, ulceration, and cancer, *Helicobacter pylori* seems to be a quite common causative agent. It is a Gram-negative spiral or curved rod-shaped bacillus with a preference for microaerophilic environments. As estimated by various researchers, almost half the world’s population harbors *H. pylori* [14,17]. In most people, it exists as part of the normal flora, but in others, it causes severe gastrointestinal illness as gastritis and ulcers, and thus, it is considered as a significant cause of morbidity and mortality with serious implications on healthcare systems [17,18,19]. For the eradication of *H. pylori* from the stomach, the therapeutic approach includes the *per os* administration of antiulcer agents (e.g., ranitidine), protein pump inhibitors (e.g., omeprazole), and antibiotics (e.g., metronidazole, clarithromycin, or amoxicillin) [20]. A major problem associated with this approach is its increased rate of failure due to the increased resistance of this microorganism to the antibiotics used despite the initial sensitivity tests of the implicated isolates [21,22]. A possible solution for milder treatments with fewer side effects for the eradication of *H. pylori* could be the natural honey, which is reported to inhibit the growth of the microorganism in a concentration of 20%. Similar findings against *H. pylori* have also reported about particular types of honey (e.g., Manuka) through in vitro experiments [23,24,25].

This study intended to survey the potential antibacterial activity of the wild Greek oregano honey against some clinical isolates of *H*. *pylori*, expanding the therapeutic use of honey and thus consolidating the medicinal properties of wild honey type as a possible new therapy for *H. pylori* eradication.

## 2. Materials and Methods

In total, 56 raw unprocessed honey samples, produced by bees bred in oregano fields of alpine altitudes at the mountainous area of Epirus, Greece, were received directly from 56 different local producers. The identification of the botanical source of each honey sample was performed by the providers based on the flora availability during the harvest season, the location of the apiary, and, in some cases, pollen analysis. A unique reference number was assigned to each sample and details regarding the botanical source, geographical location, and date of harvest were recorded. The samples, 1000 mL each, were collected in jars of glass (one jar from every producer), did not contain any additives or diluents, and had not been heated. They were evaluated for their microbiological quality by being dissolved in cation-adjusted Mueller–Hinton broth (CAMHB; Oxoid, Ltd., Basingstoke, Hampshire, UK) and subsequently inoculated into blood agar (Columbia agar base with 5% sheep blood, Becton Dickinson, MD, USA), Egg Yolk Agar (EYA, BBL™, Becton, Dickinson, MD, USA) and Mannitol–egg yolk–polymyxin agar (MYP; Oxoid, Ltd., Basingstoke, Hampshire, UK). All samples were incubated aerobically at 37 °C for 48 h and samples with evidence of bacteria growth or growths of more than 5 yeast colonies were excluded. Thus, 6 samples were excluded from our study, leaving 50 eligible samples, which were stored at 5 °C in dark glass vials to prevent photo degradation.

### 2.1. Microbial Cultures and Antibiotic Sensitivity of H. pylori Strains

In thisstudy,14clinical isolates of *H. pylori* were used. Six (6) were isolated from gastric ulcers (*Hp*U1–*Hp*U6) and eight (8) from cases of clinical gastritis (*Hp*G1–*Hp*G8). *H. pylori* DSM21031 was used as a reference strain. The clinical isolates of *H. pylori* were cultivated on Campylobacter agar (BBL™, Becton, Dickinson, MD, USA)and Columbia blood agar base (Oxoid, Ltd., Basingstoke, Hampshire, UK) plates enriched with 10% laked horse blood. Incubation was performed for 5 days at 37 °C in microaerophilic conditions (10% CO_2_, 10% O_2_, and 80% N). Bacteria cells (colonies) that appeared on the agar plates were Gram stained and examined for their morphology (e.g., spiral shape), as well as for oxidase and urease activities [26,27,28]. Spiral shaping, urease, and oxidase-positive colonies were considered typical *H. pylori* strains.E-test strips for *H. pylori* (Biomerieux; Basingstoke, UK) were used to determine the antibiotic susceptibility of the above clinical isolates and reference strain. The antibiotics used were amoxicillin, clarithromycin, metronidazole, tetracycline, levofloxacin, and rifampicin. The strains were categorized as resistant or sensitive according to the EUCAST (European Committee on Antimicrobial Susceptibility Testing) breakpoints [29,30,31,32].

### 2.2. Determination of Antibacterial Activity of Native Oregano Honey

Different concentrations of samples were prepared aseptically with the aid of sterile saline solution: 75%, 50%, 25%, 12.5%, and 6%. The agar well diffusion assay was employed to screen for the antibacterial activity of honey samples according to the method proposed by Al-Somal [24]. In brief, a standard suspension of 0.5 McFarland units from each examined *H. pylori* strain was prepared, and then an amount of 100 μL was evenly spread on Columbia blood agar base (Oxoid, Ltd., Basingstoke, Hampshire, UK) plates containing 10% laked horse blood by using a sterile cotton swab, and the plate was allowed to dry for 10 min. Following inoculation, 6.2 mm diameter wells were opened into the agar by using a sterile cork borer. An amount of 50 μL of the tested honey sample was loaded to the well, and the plates were incubated in microaerophilic conditions (10% CO_2_, 10% O_2_, and 80% N) at 37 °C for 72 h. The antibacterial activity was estimated after measuring the diameter of the inhibition zones that appeared around the wells. Samples of honey with zones of inhibition of 8mm and above in diameter were then double diluted (1:2 and up to 1:8) in order to obtain the final endpoints. The procedure was repeated twice with two plates per concentration of honey tested. A sterile dH_2_O solution served as a negative control, while for the positive control, a clarithromycin disk (15 μg) was used.

#### Osmotic Effect

A sample of 75% sucrose (*w*/*w*), corresponding to the amount of sugar in honey, was made by diluting 7.5 g sucrose in 2.5 g sterile Milli-Q water (Milli-Q^®^Reference Water Purification System, Merck, Germany). A sample of 15% sucrose (*w*/*w*), corresponding to the amount of sugar in 20% honey samples, was made by diluting 1.5 g sucrose into 8.5 g sterile Milli-Q water. Then, 50 μL of the pure sugar samples was placed in the agar wells, and the procedure followed as described above, in Section 2.2.

The pure sugar samples (75% and 15% sucrose) did not show any inhibition on the tested *H. pylori* strains.

### 2.3. Study Design for Determination In Vitro Anti-Helicobacter pylori Activity of Extracts of Crude Honey

#### 2.3.1. Used Solvents

n-Hexane [CH(CH₂)₄CH₃; for analysis EMSURE^®^ ACS, Merck, Germany], diethyl ether [CH_3_CH_2_)_2_O; for analysis EMSURE^®^ ACS, Merck, Germany], ethyl acetate (CH_3_COOC_2_H_5_; for analysis EMSURE^®^ACS, ISO, Reag. Ph Eur, Merck, Germany), chloroform (CHCl_3_; for analysis EMSURE^®^ACS, ISO, Reag. Ph Eur, Merck, Germany) and distilled water were used. The aforementioned solvents of extractions were arranged according to the order of increasing polarity, such as n-hexane (0.009 polarity), which is the least polar solvent, to water, which is the most polar (1.000 polarity).

#### 2.3.2. Extraction of Crude Honey

For the assessment of the antibacterial potency of the different types of extraction of crude honey samples, on the basis of polarity, we used the *Separation funnel method* (physical method), starting by the complete dissolution of crude honey (100 g) with 150 mL of sterile distilled water (base hydrolyze), by placed them into a sterile sample bag and mixed well using the mechanical Stomacher device. This is followed by transfer into a 500 mL separating funnel, shaken, and allowed to settle. Subsequently, 50 mL of n-hexane, the least polar solvent, was added and shaken. The shaking time for each extraction process was 15 min, after which the mixture was allowed to stand to permit the solvent layer to separate. Then, the bottom of the separating funnel opened to remove the aqueous layer. The remaining content in the separating funnel was poured into a clean container to obtain *n*-hexane fraction. An equal volume of *n*-hexane was added again, shaken, and separated. The addition continued until three successive extractions were achieved. A similar cycle was performed for diethyl ether, ethyl acetate, and chloroform, to obtain diethyl ether, ethyl acetate, and chloroform fractions, respectively. The mixture of the three successive layers was collected and mixed well, and water contaminating extracts were removed by filtration over anhydrous sodium sulfate. Finally, the collected organic solvent extract was concentrated by evaporating the extract under reduced pressure using a rotary evaporator (KNF RC 900, KNF Neuberger GmbH, Breisgau, Germany) at 40 °C, 30 °C, 60 °C, and 50 °C for *n*-hexane, diethyl ether, ethyl acetate, and chloroform, respectively [33,34].

#### 2.3.3. Screening the Antibacterial Efficacy of Honey Extracts

##### Antimicrobial Assay

In vitro antibacterial activity of honey extracts of n-hexane, diethyl ether, chloroform, and ethyl acetate were evaluated using the agar well diffusion assay, as described for crude honey in Section 2.2. Since all oregano honey samples were most active at 75% *v*/*v* concentration, accordingly, the different extracts of the most active honey at 75% concentration were tested against the strains of *H. pylori.* The positive (standard antibiotic clarithromycin disk; 15 μg/mL) and negative (pure solvent; *n*-hexane, diethyl ether, chloroform, and ethyl acetate) controls were examined by the same procedure. Diameters of zones of inhibition of each honey extract were measured; averaged and mean values were recorded in millimeters. The solvent control revealed no activity.

##### Minimum Inhibitory Concentration (MIC)

The minimum inhibitory concentration (MIC) of the four extracts from crude honey samples was determined using the broth microdilution method in 96-well microtiter plates, using the method of Manyi-Loh et al., 2010 [34]. The 96-well microplate consists of 12 columns and 8 rows. Initially, all wells were supplemented with 100 µL of brain–heart infusion broth (BHI; Oxoid, Ltd., Basingstoke, Hampshire, UK) except for Column 12, which was supplemented with 200 µL of ΒHI and used as a sterility control. Column 11 was supplemented with 100 µL of bacterial suspension and used as the growth control. Two-fold dilutions were prepared in test wells in ΒHI broth (BHI; Oxoid, Ltd., Basingstoke, Hampshire, UK); the final honey extract concentration was 0.001–12% *v*/*v*. The six (6) isolates of *H. pylori* recovered from patients from gastric ulcers (*Hp*U1–*Hp*U6) were selected for the determination of MICs. The inoculum of each mentioned strain was diluted tenfold in a sterile saline solution. Then, a volume of 20 μL of the bacterial suspension 1 × 10^8^colony-forming units [CFUs]/mL, corresponding to 0.5 McFarland standards, was added to each well. All the test plates were sealed and incubated at 37 °C under microaerophilic conditions for 72 h. After the period of incubation, 32 μL of resazurin was added per well, coloring them blue. Subsequently, the plates were entered for incubation for an additional 1 h, and the growth of bacteria in broth was observed by visual inspection and by measuring optical density (OD) using an ELISA microplate reader (ELx808™(BioTek Instruments, Inc., Bad Friedrichshall, Germany) at 620 nm. The MIC was defined as the lowest concentration of honey extract that prevented resazurin color change from blue to pink. Similarly, at the ELISA microplate reader, it was the lowest concentration of the honey extract that resulted in inhibition of bacterial growth by 95%.

### 2.4. Urease Inhibitory Effect

Urease inhibitory effect was assayed via the alkalimetric method, according to Hamilton Miller and Gargan (1979), Mobley et al. (1988), and Korona-Glowniak et al. (2020) for urease preparations of *H. pylori* DSM21031, *Hp*U1, and *Hp*U2 strains in the presence of potential urease inhibitors [35,36,37]. In a 96-well plate, a volume 10 µL of bacterial suspension with a density of three (3) according to the McFarland scale, (approximately; 3 × 10^8^ cells (CFU)/1 mL) was mixed with various concentrations of inhibitors (a: crude honey, as described below; b: honey extracts with the solvent diethyl ether), in urea medium (0.1 g/L yeast extract, 9.1 g/L KH_2_PO_4_, 9.5 g/L Na_2_HPO_4_, 20 g/L urea, and 0.01 g/L phenol red) and the assay cocktail mixed thoroughly by pipetting. After 24 h of incubation at 35 °C in microaerophilic conditions, absorbance was measured at 560 nm. The concentration of the inhibitor required to diminish enzyme activity by approximately 100% was calculated by plotting percent inhibition against the concentration of inhibitor. As a reference value, 100% activity was determined in the absence of an inhibitor.

*Clarification*:

(a) Crude honey

Approximately 10 g of the honey sample was extracted with 50 mLof distilled water in a flask attached to a condenser at 60 °C for more than 6 h. The extract was subsequently filtered to remove particles, and the final volume was adjusted with an ultrapure water solution.

(b) Dried honey extracts with the solvent diethyl ether

Dilution of dried honey extracts for the MIC experiment: Totally dried honey extracts were weighed, and a 400 mg/mL honey/ultrapure water solution was prepared and kept for 24 h before it vortexed at 1500 rpm for 3 min and filtered through a 0.45 Whatman TM syringe filter (Merck, Germany) from which serial dilutions were performed to produce subsequent concentrations, i.e., 200 mg/mL, 100 mg/mL, 50 mg/mL, 25 mg/mL, 12.5 mg/mL, 6.25 mg/mL, 3.125 mg/mL, 1.56 mg/mL, 0.78 mg/mLand 0.39 mg/mL.

### 2.5. Statistical Analysis

Analysis of variance with Tukey’s HSD *post hoc*comparison was used to compare more than two groups of samples, while in the case of two groups of samples, the Mann–Whitney (Wilcoxon) nonparametric test was applied. Spearman Rho correlation coefficient was estimated when the degree of correlation was required. All analyses were performed with SPSS v25 (IBM Corp. 2017. Armonk, NY, USA) at an alpha of 95%.

## 3. Results

The results of the antibacterial activities of the various Greek oregano honey samples in the present study are presented in Table 1 (see supplementary material). In general, the reference strain *H. pylori* DSM21031 and various gastritis strains proven to be the most sensitive ones since their average inhibition zones were among the widest observed for 75% (*v*/*v*) concentration of honey. However, there were statistically significant differences among them since in three out of the eight gastritis strains (*Hp*G2, *Hp*G3, *Hp*G7), the inhibition zones were particularly large and over 50 mm in diameter. In contrast, the strains isolated from ulcers were almost more resistant to 75% (*v*/*v*) honey concentration, with average inhibition zones ranged from 15.38 ± 3.3 mm to 29.1 ± 5.1 mm. In this case, there were no statistically significant differences, and the six ulcer strains behaved as a more homogenous group when compared with those from gastritis. There was a similar profile when the *H. pylori* strains were treated with a 50% (*v*/*v*) concentration of honey. Ulcer strains were more resistant than the gastritis strains since the first exhibited zones from 14.36 ± 3.9 to 27.15 ± 4.0 mm in contrast with 32.13 ± 3.9 mm to 45.88 ± 4.0 mm of the latter. The inhibition exhibited by the reference strain was somewhere between (30.24 ± 1.5 mm). The group of the ulcer strains remained homogenous and in that honey concentration, and this was also the case when lower concentrations of honey tested (25%, 12.5%, and 6% *v*/*v*) something also apparent from the minimum values of inhibition zones recorded. Those particular strains were proven also to be multiresistant to the synthetic drugs since in 4 out of the 6 antibiotics, they exhibited larger minimum inhibitory concentration (MIC) values than indicated by the clinical breakpoints (Table 2).

In general, the strains of *H. pylori* tested could be arranged in order as the ulcer strains being the least susceptible followed by the reference and the gastritis strains.

**Figure 1:**
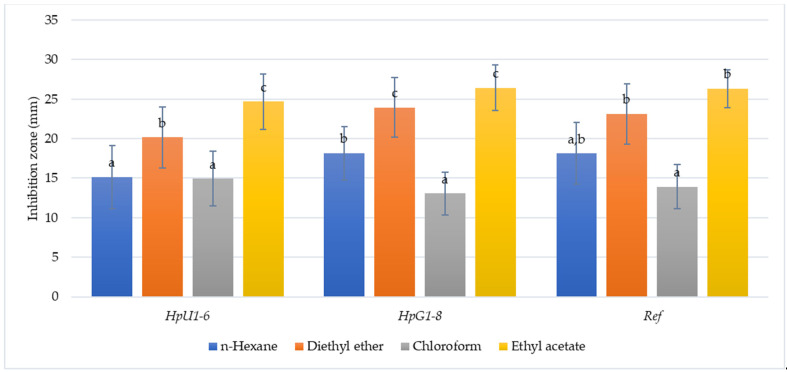
Mean diameter of inhibition zones from various solvent extracts against ulcer (*Hp*U1–6, *n* = 6), gastritis (*Hp*G1–8, *n* = 8), and the reference strain of *H. pylori* DSM21031. Different letters in bars indicate statistically significant differences (ANOVA with Tukey’s HSD, *p* < 0.05) for each group of strains.

The activities of 50 Greek oregano honey extracts against 14 *H. pylori* strains, as recorded from the diameter of their inhibition zones (mean values ± standard deviation), are presented in Table 3 and summarized in Figure 1. On average, inhibition zones of *n*-hexane extracts in various strains ranged from 12.85 ± 6.4 mm to 19.7 ± 4.2 mm and 18.13 ± 3.9 mm for the reference strain *H. pylori* DSM21031, with no statistically significant differences (ANOVA F:0.59, *p* > 0.05) among them. Inhibition zones from diethyl ether extracts ranged from 19.5 ± 3.7 mm to 26.4 ± 3.1 mm and 23.12 ± 3.8 mm for the reference strain (ANOVA F:0.87, *p* > 0.05). Inhibition zones from chloroform extracts ranged from 12.1 ± 2.1 mm to 15.78 ± 5.2 mm, and those from ethyl acetate from 23.78 ± 4.7 mm to 27.8 ± 1.8 mm, with 13.9 ± 2.8 mm and 26.35 ± 2.4 mm being the respective values for the reference strain. Again, no statistically significant differences were noted among the various as well as the reference strain (ANOVA F:0.27, *p* > 0.05 and F:0.34, *p* > 0.05). However, differences in inhibition zones were recorded within each strain regarding its sensitivity in various solvent extracts. As indicated in Table 3, such differences were observed for strain *Hp*U6 (ulcer), all gastritis strains (*Hp*G1–*Hp*G8), and the reference, in which the *n*-hexane and chloroform extracts repetitively produced smaller inhibition zones, in contrast to the ethyl acetate and diethyl ether extracts, and thus, increased susceptibility to the latter was signified. Based on our results, diethyl ether extracts of oregano honey produced inhibition zones 27–32.8% larger than those of *h*-hexane and 34.8–83.4% larger than those of chloroform, while inhibition zones from ethyl acetate extracts were larger by 45.3–62.7% and 65.5 to 102.4%, respectively (Figure 1) (see supplementary material). The diameter of the inhibition zones was positively correlated between *n*-hexane and diethyl ether extracts (Spearman *r* = 0.45, *p* < 0.05) and negatively correlated between chloroform and diethyl ether extracts (Spearman *r*= −0.42, *p* < 0.05).

**Figure 2:**
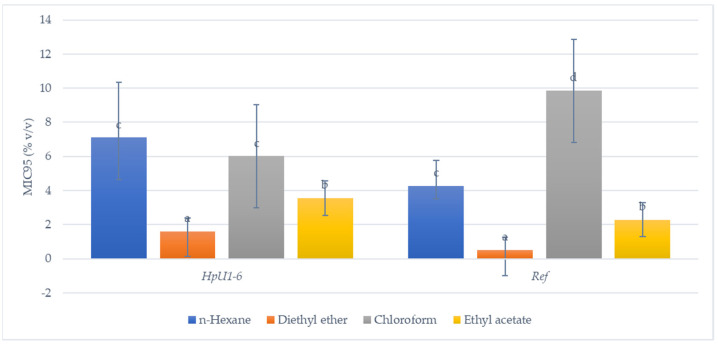
MIC_95_ values (% *v*/*v*) of *h*-hexane, diethyl ether, chloroform, and ethyl acetate oregano honey extracts against (6) *H. pylori* clinical strains (*Hp*U1–*Hp*U6) isolated from ulcers and a reference strain (*H. pylori* DSM210321). Different letters above bars indicate statistically significant differences (ANOVA with Tukey’s HSD, *p* < 0.05) for each group of strains.

As already mentioned above, MIC_95_ values (95% bacterial growth inhibition) of the four solvent extracts (% *v*/*v*) against *H. pylori* ulcer strains, as well as against the reference strain DSM21031, were estimated. Out of 1400 MIC tests, 53.8% (754) showed an inhibitory effect against the various strains, and their results are presented in Table 4. Overall, mean MIC_95_values ranged from 0.52 ± 0.76% (*v*/*v*) to 10.23 ± 2.25% (*v*/*v*), indicating increased variations between the various experiments. However, lower MIC_95_ values (i.e., an increased inhibitory action) were observed in all strains for ethyl acetate and diethyl ether extracts (Figure 2), which complies with the inhibition zone experiments already presented. Oregano honey extracts with n-hexane as solvent exhibited MIC_95_ values ranging between 4.28 ± 2.45% (*v*/*v*) and 10.28 ± 2.25% (*v*/*v*), while their respective ranges for chloroform, ethyl acetate and diethyl ether extracts were 4.26 ± 2.36% (*v*/*v*) to 9.85 ± 2.27% (*v*/*v*), 2.29 ± 1.25% (*v*/*v*) to 4.82 ± 2.03% (*v*/*v*), and 0.52 ± 0.76% (*v*/*v*) to 3.11 ± 2.84% (*v*/*v*), respectively. It is of note that in these experiments, the lowest MIC_95_ values were observed when diethyl ether was used as a solvent, while the largest inhibition zones were recorded with ethyl acetate extracts in the previous ones. Additionally, in these experiments, *H. pylori* DSM21031 strain proved to be among the most sensitive in all but the chloroform extracts, while no such differences were noted when the inhibition zones accounted. Finally, all MIC_95_ values from the various extracts were positively correlated, but the strongest was between n-hexane and chloroform extracts (Spearman r = 0.50, *p* < 0.05).

In order to study the possible urease inhibitory effect, pure oregano honey samples and diethyl ether extracts were tested side by side for their inhibitory effectivity against two *H. pylori* strains (*Hp*U1, *Hp*U2) isolated from ulcers and the reference strain *H. pylori* DSM21031 via the method described in the Materials and MethodsSection. The effective concentrations of pure and extracted honey samples are presented in Table 5. From 50 samples, 7 (14%) pure honey extracts showed no effective concentration against HpU1, while only one (2%) behaved similarly on HpU2. Additionally, 3 (6%) honey samples had no effect used either as pure or extracted with diethyl ether as a solvent, and in one sample (2%), the effective concentration was increased. However, the majority of experiments revealed a drastic increase of effectiveness as depicted by the corresponding decrease in the effective concentration of diethyl ether extract from each honey to inhibit the growth of the particular *H. pylori* strain. In the *Hp*U1 strain, the mean effective concentration was reduced from a mean value of 91.3 ± 55.8 mg/Ml to 22.0 ± 18.8 mg/mL, which corresponded to an average decrease of 67.9%. In *Hp*U2, this decrease was 86.2% (from 57.5 ± 40.5 to 5.93 ± 3.32 mg/mL), and for the reference strain 81.5%, i.e., from 28.3 ± 18.3 to 4.43 ± 2.33 mg/mL. Wilcoxon’s sum-rank test showed that all three reductions were statistically different, as indicated in Table 5.

## 4. Discussion

Since its first isolation in 1899, *H. pylori,* has drawn attention as a causative pathological agent for gastric diseases [32,38]. The microorganism is found in gastric and duodenal mucus but also forms colonies in Barrett’s esophagus and, in this way, it is related to numerous cases of esophagitis [39,40]. The bacterium is capable to survive—for many microorganisms—in a hostile environment such as the stomach by converting (with the aid of urease) urea into ammonia and thus interacting with the stomach acid. In this way, a neutral microenvironment is created that protects the microorganism from hydrochloric acid, allowing further colonization, which leads to various gastric infections and gastric-related diseases [41]. According to various studies, a persistent infection by *H. pylori* could even favor the development of gastric cancer [42,43]. Currently, the hypothesis that *H. pylori* infections may cause cancer is strongly debated, making *H. pylori* a candidate for the first bacterium recognized as a class I carcinogen [44].

In contemporary clinical practice, single, or multidrug regimens are the only accepted treatment for *H. pylori* eradication. However, unsuccessful eradication of *H. pylori* infections and recurrent infections due to increasing resistance of *H. pylori* to therapeutic treatments with antimicrobials agents, have been reported (it is estimated that 5–20% of treatments fail) [44,45,46,47,48]. Even when the use of antibiotics as first-line therapeutic agents was the appropriate approach for ordinary cases of *H.pylori,* many reports have shown that often a multiresistant strain was involved [45,46,47,48]. All these have led to ongoing research for alternative treatments aiming at *H. pylori*’s eradication.

A nonconventional medical treatment that has recently received much attention is honey. Honey is produced by honeybees of the genera *Apis mellifera* and *Meliponinae*, which graze various sources. Its antimicrobial activity varies greatly with its origin and its processing and is also dependenton the natural vegetative flowers blooming in different seasons and in different places [49,50]. Honey has also anti-ulcerous properties because it contains flavonoids, which increase the prostaglandin content of the gastric mucosa and inhibit acid secretions [51]. For example, hesperitin and naringin are flavonoids originating from orange blossoms [52]. Various researchers argue that these flavonoids, acting in synergy with other compounds such as esterols, terpinens, and saponins, which are also found in honey [53,54,55,56].

Considering the antibacterial properties of honey, they have been attributed to factors such as the low pH, osmotic effect, high sugar concentration, presence of bacteriostatic or bactericidal substances (hydrogen peroxide, various antioxidants, enzymes as lysozyme, polyphenols, phenolic acids, flavonoids, methylglycoxal) and even to various bee peptides [1,57]. Researchers ascribe the antibacterial action of honey against Gram-positive bacteria to aromatic acids and esters, while others report that pinocembrin, galangin, and caffeic acid phenyl ester from honey inhibit the bacterial RNA polymerase [58,59]. Certain flavonoids induce severe damage to the cytoplasm membrane of the bacteria, leading to cell autolysis [60]. Quercetin is another honey compound that alters membrane permeability, thus disrupting its electrical potential and lowering ATP synthesis [61]. Bradzynski [62] argues that the antibacterial compounds of honey act in modes such as β-lactamic antibiotics, antibacterial peptides, or inhibitors of proton motive force.

Oregano (*Origanum vulgare*) is an aromatic, medicinal, and culinary plant known for its strong antioxidant properties. It contains various bioactive compounds that participate in the chemotype of the oregano essential oil such as rosmarinic acid, thymol, and carvacrol. All these compounds, as well as others (flavonoids, triterpenoids, sterols, vitamin C, and vitamin A), have been considered responsible for their various beneficial (anti-inflammatory, antibacterial, antioxidant, antifungal, and antiviral) properties. Such antibacterial properties able to treat infections of the stomach and various digestive disorders have been demonstrated by recent studies [63,64].

Part of our research is to study if these reported benefits of the oregano plant are passed into the honey produced from bees that pollinate the plants. Furthermore, a unique property that honey possesses is its relatively low pH value (approx. 3.9), and hence, its dilution by gastric juices in the stomach will activate its glucose oxidase content. According to various studies, the activated glucose oxidase liberates hydrogen peroxide with known antibacterial properties but also recognized as a compound that acts as an activator of the fibroblasts and the epithelial cells, which are required during the healing of the ulcers caused by the pathogen [65]. Although *H. pylori* thrives in acid pH, causes an infection under alkaline pH due to its ability to hydrolyze urea from the mucosa cells and liberating ammonia. The acid pH of honey can neutralize this environment, thereby facilitating the healing process [23,66,67].

In the present study, we have demonstrated in vitro that Greek locally obtained unprocessed oregano honey may be active against *H. pylori*. In fact, our data show that all the 50 honey samples tested exerted some antibacterial activity, although with considerable fluctuation, probably due to the differences in the chemotype of the aromatic plants, which are chemically diversified in compositions even among members of the same plant species. These fluctuations could rely on the presence of additional natural components as bees are able to collect the nectar from different sources. Additionally, other environmental, climatic, and farming factors can influence the chemotype of oregano plants [68].

Although raw oregano honey shows potential for use as an innocuous agent against *H. pylori*, it is important to study this activity further since promising in vitro results have been proven ineffective in clinical trials [25]. The extracts from oregano honey were recovered with the aid of various organic solvents. However, these solvents do not have the same polarity; instead, their polarity is in ascending order: n-hexane, diethyl ether, chloroform, and ethyl acetate. These differences in polarity determine the concentrations and the chemical species, which pass to the extract during the extraction process, meaning that solvents with different polarities will extract different mixtures of compounds and hence the differences observed in the results of Table 3.

The origin of the clinical *H. pylori* strains used in the present study is an important factor because it shows a multivariant profile, as different clinical strains are associated with different genotypes. Our findings are in accordance with the ones of other researchers who, in vitro, found that different types of honey possess antibacterial activity against *H. pylori* [24,69,70,71].

The basic reason for which the strains originating from ulcers were selected is that such strains are more aggressive or more virulent than the ones causing gastritis [72]. Our results show that the ulcer stains were, in general, more resistant to the antibiotics than the gastritis ones, as shown in Table 2, and particularly in amoxicillin and in clarithromycin. Both substances are the medicines of choice in the clinical practice for the treatment of *H. pylori* infections. For most antibiotics, the strains originating from ulcers were found more resistant than the ones originating from gastritis except for metronidazole and tetracycline. The latter, although it has been used in clinical practice, it is not considered a medicine of choice, while resistance to metronidazole is also reported by other researchers.

The observed differences in the rows of Table 3 imply the difference in susceptibility among these strains and is a rather expected finding. The differences in the columns show the different impacts of the different extracts on the growth of every strain. For all strains, chloroform and *n*-hexane extracts showed the largest inhibition zones. Strains *Hp*U1 and *Hp*U2 showed a significantly larger inhibition zone due to the n-hexane extract, in comparison to the chloroform extract. In the other strains, these differences were not statistically significant, except for the reference strain, where the chloroform extract showed a significantly larger inhibition zone. Diethyl ether, although the most polar solvent of all, showed smaller inhibition zones for all strains; this suggests that the compounds with antibacterial action in oregano honey have a moderate polarity. This finding could be useful in future studies on the nature of these substances. Figure 2 illustrates the same results as MIC values, and it can be clearly seen that n-hexane extract and chloroform extracts possess the most potent antibacterial activity.

The antibacterial effect of honey has been attributed, to some degree, to the osmotic effect caused by the high concentration of sugars. For comparison purposes, artificial honey was prepared in two concentrations (75% sucrose and 15% sucrose). The results showed no significant inhibitory result on the *H. pylori* strains. This finding shows a resistance of this bacterium to the hyperosmotic effect, and we believe that it underlies an evolutionary defense mechanism of survival in the micro-hyperosmotic environment caused in the stomach by foods containing, e.g., too much sugar or too much salt. The results presented in Table 1 show that the more diluted the honey is, the smaller the diameters of the inhibition zones are. The most valid interpretation of these findings—after negating the osmotic effect—is that the chemicals with antibacterial activity are also diluted, hence the smaller inhibitory zones.

A very characteristic trait of *H. pylori* is the hydrolyzation of urea to ammonia with the aid of the enzyme urease. The mucosal secretions of the stomach contain urea and by evolutionary adaptation, *H. pylori* converts it to ammonia, which reacts with hydrochloric s\acid, and thus, a neutral microenvironment is created around the bacterium. If the action of urease is inhibited, then the bacterium is no longer protected from acidic–gastric secretions. To investigate the possible inhibition of urease by the oregano honey, a set of experiments were performed, as described in the Materials and Methods Section. In short, two *H. pylori* strains, isolated from ulcers and the reference strain, were exposed to dry honey and to the diethyl ether extract, and the effective concentrations in which urease was inhibited were measured. The results indicate that urease secretion was inhibited in all tested strains, while the effective concentrations of the diethyl ether extracts were significantly lower than the ones of the crude honey. It is obvious that the diethyl ether extracts of oregano honey managed a higher concentration of the responsible chemical factors, compared to the crude oregano honey, which inhibits/suspends the urease secretion.

## 5. Conclusions

The antibacterial properties of honey have been thoroughly studied during the last years. However, most of these studies were conducted in non-European countries related to honey samples of different origins. In this study, we attempted to assess the value of oregano honey as an antimicrobial agent against *H. pylori.* Oregano honey showed a prominent antimicrobial profile and, to some extent, justifies its use by the locals as a remedy to treat empirically gastric ailments. The conclusions of our study can be summarized as follows:

1. All strains of *H. pylori* were susceptible to the action of oregano honey, although not all of them equally.
2. The *H. pylori* strains were tested for their susceptibility against sixantibiotics that are used in clinical therapeutics to treat *H. pylori* infections, and the results indicate that, in general, the strains that were isolated from ulcers were more resistant than the ones isolated from gastritis cases.
3. The extracts of oregano honey by the four different organic solvents showed significant differences in their antibacterial activity against *H. pylori*. Each of these solvents, for chemical reasons, extracts a different mixture of compounds, and this finding points to a synergistic effect of these compounds rather than to one super drastic compound with antibacterial effects.
4. *H. pylori* was found to be resistant to artificial honey (sucrose 75% and 15%), implying resistance to the osmotic effect.
5. Oregano honey inhibits the activity of urease secreted by *H. pylori*, rendering it vulnerable to the acidic pH of the stomach.
6. Future perspectives include the chemical identification of the substances responsible for the antibacterial effect of oregano honey as well as conducting clinical trials for volunteers.

## Figures and Tables

**Table 1:** Antibacterial activity (zone of inhibition in mm) of oregano honey samples against *H. pylori* strains isolated from clinical samples (ulcers = 6, gastritis = 8) and a reference strain (*H. pylori* DSM21031).

Strain	Concentration of Honey (% *v*/*v*)				
	75	50	25	12.5	6
*Hp*U1	27.26 ± 6.4 ^ab^	22.28 ± 6.3 ^abc^	15.2 ± 6.9 ^ab^	6.56 ± 3.4 ^a^	3.16 ± 1.7 ^a^
*Hp*U2	21.45 ± 5.1 ^ab^	18.63 ± 4.14 ^ab^	13.8 ± 3.9 ^a^	7.7 ± 3.1 ^a^	2.41 ± 1.28 ^a^
*Hp*U3	15.38 ± 3.3 ^a^	14.36 ± 3.9 ^a^	12.18 ± 2.9 ^a^	9.18 ± 2.2 ^ab^	5.14 ± 2.1 ^ab^
*Hp*U4	22.48 ± 4.81 ^ab^	18.16 ± 3.56 ^ab^	13.3 ± 3.45 ^a^	9.19 ± 3.39 ^ab^	3.18 ± 1.2 ^a^
*Hp*U5	29.1 ± 5.1 ^ab^	21.45 ± 3.1 ^abc^	18.13 ± 4.3 ^abc^	5.48 ± 4.1 ^a^	3.5 ± 1.8 ^a^
*Hp*U6	28.78 ± 4.18 ^ab^	27.15 ± 4.0 ^bcd^	21.45 ± 3.6 ^abc^	18.13 ± 1.3 ^cd^	12.14 ± 2.4 ^bc^
*Hp*G1	47.7 ± 4.5 ^cd^	43.5 ± 4.7 ^h^	36.26 ± 3.7 ^d^	30.44 ± 1.6 ^e^	20.1 ± 2.9 ^d^
*Hp*G2	50.28 ± 4.2 ^d^	45.88 ± 4.0 ^h^	26.66 ± 4.6 ^bcd^	15.7 ± 3.9 ^abc^	5.48 ± 5.4 ^ab^
*Hp*G3	53.44 ± 3.8 ^d^	32.13 ± 3.9 ^cdeg^	14.44 ± 2.14 ^a^	9.90 ± 1.6 ^ab^	3.78 ± 2 ^a^
*Hp*G4	35.16 ± 4.13 ^abc^	28.76 ± 3.28 ^bcde^	18.90 ± 4.2 ^ac^	13.12 ± 2.45 ^abc^	4.13 ± 1.1 ^a^
*Hp*G5	44.6 ± 5.51 ^cd^	42.42 ± 4.5 ^egh^	34.66 ± 3.9 ^bd^	27.24 ± 4.4 ^de^	18.00 ± 4.0 ^acd^
*Hp*G6	48.84 ± 5.3 ^cd^	33.18 ± 3.64 ^cdeg^	29.88 ± 4.18 ^cd^	22.14 ± 4.4 ^ce^	15.70 ± 3.15 ^cd^
*Hp*G7	51.51 ± 5.8 ^cd^	40.28 ± 4.6 ^egh^	27.80 ± 4.3 ^cd^	12.90 ± 3.45 ^abcd^	4.15 ± 2.1 ^a^
*Hp*G8	47.62 ± 3.9 ^ad^	35.66 ± 4.9 ^degh^	29.22 ± 4.3 ^cd^	15.78 ± 3.5 ^abc^	4.6 ± 1.8 ^ab^
Ref	51.24 ± 2.4 ^d^	30.24 ± 1.5 ^bcdeg^	15.4 ± 1.1 ^ab^	10.8 ± 1.3 ^abc^	1.34 ± 1.1 ^a^

*Hp*U1 to *Hp*U6 are *H. pylori* strains isolated from ulcers, while *Hp*G1 to *Hp*G8 are *H. pylori* strains isolated from gastritis. Ref: *H. pylori* DSM21031 (reference strain). Similar superscript letters indicate no statistically significant differences (ANOVA with Tukey’s HSD *post hoc* multiple range tests) among the various *H. pylori* strains for the same concentration of honey.

**Table 2:** Antibiotic susceptibility results from six antibiotics against *H. pylori* strains isolated from clinical samples (ulcers = 6, gastritis = 8) and a reference strain (*H. pylori* DSM21031).

Antibiotic	*Hp*U1	*Hp*U2	*Hp*U3	*Hp*U4	*Hp*U5	*Hp*U6	*Hp*G1	*Hp*G2	*Hp*G3	*Hp*G4	*Hp*G5	*Hp*G6	*Hp*G7	*Hp*G8	*Hp*Ref	*Clinical Breakpoints* (mg/L) *	
																Susceptible	Resistant
Amoxicillin	R	R	S	R	R	R	S	S	S	S	R	S	R	S	S	≤0.12	>0.12
Clarithromycin	R	S	R	S	R	R	R	S	S	S	S	S	S	S	S	≤0.25	>0.5
Metronidazole	S	R	R	S	S	R	S	S	R	R	S	S	R	R	S	≤8	>8
Tetracycline	R	S	S	R	R	S	S	S	S	R	R	R	R	S	S	≤1	>1
Levofloxacin	R	R	R	S	R	S	S	R	R	S	R	S	S	S	S	≤1	>1
Rifampicin **	R	S	R	R	S	R	S	S	R	S	R	S	S	S	S	≤1	>1

* Proposed by EUCAST (European Committee on AntimicrobialSusceptibility Testing) and the British Society for Antimicrobial Chemotherapy. ** Rifampicin was used to screen for rifabutin resistance since rifampicin E-tests are not available routinely (adapted from EUCAST). R: Resistant, S: Sensitive.

**Table 3:** Diameter of the inhibition zones (mm, mean ± StDev) produced from various solvent extracts (75% *v*/*v*) against 14 *H. pylori* strains isolated from ulcers (*n* = 6) or gastritis (*n* = 8) and a reference strain *H. pylori* DSM21031.

Honey Extract (75% *v*/*v*)	*Hp*U1 *	*Hp*U2	*Hp*U3	*Hp*U4	*Hp*U5	*Hp*U6	*Hp*G1	*Hp*G2	*Hp*G3	*Hp*G4	*Hp*G5	*Hp*G6	*Hp*G7	*Hp*G8	*H. pylori* DSM21031
**n-Hexane**	14.35 ± 3.5 ^a^	12.85 ± 6.4 ^a^	14.89 ± 5.3 ^a^	16.41 ± 3.8 ^a^	15.74 ± 3.8 ^a^	16.81 ± 3.25 ^a^	17.38 ± 3.8 ^ab^	18.71 ± 4.5 ^ab^	17.99 ± 3.7 ^ab^	16.9 ± 3.5 ^ab^	18.45 ± 4.3 ^ab^	17.85 ± 3.6^.b^	18.4 ± 3.8 ^ab^	19.7 ± 4.2 ^ab^	18.13 ± 3.9 ^ab^
**Diethyl ether**	22.1 ± 5.7 ^a^	19.5 ± 3.7 ^a^	19.8 ± 4.7 ^a^	19.88 ± 5.3 ^a^	19.8 ± 2.9 ^a^	19.87 ± 3.7 ^ab^	23.7 ± 5.4 ^ab^	25.1 ± 4.7 ^b^	22.35 ± 4.7 ^ab^	25.8 ± 4.7 ^bc^	26.4 ± 3.1 ^b^	22.7 ± 2.8 ^ab^	22.78 ± 3.8 ^b^	22.74 ± 2.7 ^b^	23.12 ± 3.8 ^b^
**Chloroform**	14.7 ± 5.7 ^a^	15.4 ± 2.8 ^a^	14.18 ± 2.9 ^a^	15.1 ± 3.9 ^a^	15.78 ± 5.2 ^a^	14.5 ± 2.8 ^a^	12.1 ± 2.1 ^a^	13.24 ± 3.4 ^a^	12.7 ± 3.8 ^a^	13.5 ± 3.8 ^a^	12.8 ± 2.8 ^b^	13.7 ± 3.8 ^a^	12.75 ± 2.8 ^a^	13.7 ± 2.8 ^a^	13.9 ± 2.8 ^a^
**Ethyl acetate**	25.42 ± 4.7 ^a^	23.78 ± 4.7 ^a^	24.58 ± 3.5 ^a^	23.85 ± 4.8 ^a^	25.45 ± 3.8 ^a^	25.05 ± 2.7 ^b^	24.78 ± 6.1 ^b^	27.35 ± 2.1 ^b^	25.78 ± 2.5 ^b^	27.8 ± 1.8 ^c^	25.78 ± 3.2 ^b^	26.58 ± 3.5 ^b^	26.85 ± 2.4 ^b^	26.65 ± 2.5 ^b^	26.35 ± 2.4 ^b^

* Strains *Hp*U1–*Hp*U6 isolated from ulcers and *Hp*G1–*Hp*G8 from gastritis. Different superscript letters in columns indicate statistically significant differences (ANOVA with Tukey’s HSD *post hoc* comparison, *p* < 0.05) in the diameter of the inhibition zone between the solvent extracts within each *H. pylori* strain.

**Table 4:** MIC_95_ of solvent extracts (% *v*/*v*) of each honey against *H. pylori* strains isolated from ulcers and the reference strain DSM21031.

Solvent	N *	*Hp*U1	N *	*Hp*U2	N *	*Hp*U3	N *	*Hp*U4	N *	*Hp*U5	N *	*Hp*U6	*N* *	Hp DSM21031
**n-hexane**	32	10.23 ± 2.25 ^4c^	27	6.42 ± 4.16 ^3,b^	7	7.78 ± 3.55 ^3,bc^	11	5.97 ± 3.29 ^3,ab^	6	5.83 ± 3.41 ^2,3,ab^	9	6.38 ± 2.82 ^3,ab^	21	4.28 ± 2.45 ^3,a^
**Diethyl ether**	36	3.11 ± 2.84 ^1,d^	39	0.93 ± 0.96 ^1,ab^	41	2.13 ± 2.26 ^1,c^	29	0.96 ± 0.65 ^1,ab^	37	1.51 ± 1.58 ^1,bc^	46	1.02 ± 0.64 ^1,ab^	48	0.52 ± 0.76 ^1,a^
**Chloroform**	25	7.3 ± 2.78 ^3,b^	22	4.26 ± 2.36 ^2,a^	5	5.0 ± 1.76 ^2,3,ab^	12	6.83 ± 3.48 ^3,b^	13	6.21 ± 3.68 ^3,ab^	17	6.45 ± 4.07 ^3,b^	20	9.85 ± 2.27 ^4,c^
**Ethyl acetate**	28	4.82 ± 2.03 ^2,ab^	38	2.96 ± 2.51 ^2^	34	3.46 ± 3.44 ^2,abc^	33	3.35 ± 3.27 ^2,ab^	32	3.99 ± 3.24 ^2,bc^	50	2.70 ± 2.80 ^2,a^	36	2.29 ± 1.25 ^2,a^

* Number of samples with inhibitory effect out of 50 oregano honey tested samples. Different superscript numbers indicate statistically significant differences in rows while superscript numbers indicate statistically significant differences in columns (ANOVA with Tukey’s HSD *post hoc* comparisons at 95%).

**Table 5:** Urease Inhibitory effect of pure and diethyl ether extracts of oregano honey against two *H. pylori* strains isolated from ulcers (*Hp*U1, *Hp*U2) and the reference strain DSM21031.

*H. pylori* Strain	Oregano Honey	N *	Effective Concentration (mg/mL)		
			Mean ± SD	Range	Median
*Hp*U1	Pure	43	91.3 ± 55.8 ^**^	12.5–200.0	100.0
	Diethyl ether extract	50	22.0 ± 18.8	3.0–100.0	12.5
*Hp*U2	Pure	49	57.5 ± 40.5 ^**^	6.25–200.0	50.0
	Diethyl ether extract	50	5.93 ± 3.32	1.56–12.5	6.25
DSM21031	Pure	49	28.3 ± 18.3 ^**^	12.5–100.0	25.0
	Diethyl ether extract	50	4.43 ± 2.33	1.56–12.5	3.125

* Number of samples with inhibitory effect out of 50 oregano honey tested samples ** Statistically significant differences between pure and diethyl ether extract effective concentration according to the Mann–Whitney (Wilcoxon) W-test (*p* < 0.01).

## Supplementary Materials

The following are available online at https://www.mdpi.com/article/10.3390/foods10071568/s1. Table S1: Antibacterial activity (zone of inhibition in mm) of oregano honey samples against *H. pylori* strains isolated from clinical samples (ulcers = 6, gastritis = 8) and a reference strain (*H. pylori* DSM21031), Table S2: Antibiotic susceptibility results from six antibiotics against *H. pylori* strains isolated from clinical samples (ulcers = 6, gastritis = 8) and a reference strain (*H. pylori* DSM21031), Table S3: Diameter of the inhibition zones (mm, mean ± StDev) produced from various solvent ex-tracts (75% *v*/*v*) against 14 *H. pylori* strains isolated from ulcers (n = 6) or gastritis (n = 8) and a ref-erence strain *H. pylori* DSM21031, Table S4: MIC_95_ of solvent extracts (% *v*/*v*) of each honey against *H. pylori* strains isolated from ulcers and the reference strain DSM21031, Table S5: Urease Inhibitory effect of pure and diethyl ether extracts of oregano honey against two *H. pylori* strains isolated from ulcers (*Hp*U1, *Hp*U2) and the reference strain DSM21031, Figure S1: Mean diameter of inhibition zones from various solvent extracts against ulcer (*Hp*U1-6, n = 6), gastritis (*HpG1-8*, n = 8), and the reference strain of *H. pylori* DSM21031, Figure S2: MIC_95_ values (% *v*/*v*) of *h*-hexane, diethyl ether, chloroform, and ethyl acetate oregano honey extracts against (6) *H. pylori* clinical strains (*Hp*U1–*Hp*U6) isolated from ulcers and a reference strain (*H. pylori* DSM210321).
