# Supplementary material for: In Vitro Screening Potential Antibacterial Properties of the Greek Oregano Honey against Clinical Isolates of Helicobacter pylori
Source: Foods. 2021 Jul 6;10(7):1568. doi: 10.3390/foods10071568 (PMC8307090; doi:10.3390/foods10071568)
Supplement: Supplementary file 1 [file foods-10-01568-s001.zip › foods-1276519-supplementary.pdf]

foods

Table S1. Antibacterial activity (zone of inhibition in mm) of oregano honey samples against *H. pylori* strains isolated from clinical samples (ulcers = 6, gastritis = 8) and a reference strain (*H. pylori* DSM21031).

Strain	Concentration of Honey (% *v/v*)				
	75	50	25	12.5	6
*Hp*U1	27.26 ± 6.4^{ab}	22.28 ± 6.3^{abc}	15.2 ± 6.9^{ab}	6.56 ± 3.4^{a}	3.16 ± 1.7^{a}
*Hp*U2	21.45 ± 5.1^{ab}	18.63 ± 4.14^{ab}	13.8 ± 3.9^{a}	7.7 ± 3.1^{a}	2.41 ± 1.28^{a}
*Hp*U3	15.38 ± 3.3^{a}	14.36 ± 3.9^{a}	12.18 ± 2.9^{a}	9.18 ± 2.2^{ab}	5.14 ± 2.1^{ab}
*Hp*U4	22.48 ± 4.81^{ab}	18.16 ± 3.56^{ab}	13.3 ± 3.45^{a}	9.19 ± 3.39^{ab}	3.18 ± 1.2^{a}
*Hp*U5	29.1 ± 5.1^{ab}	21.45 ± 3.1^{abc}	18.13 ± 4.3^{abc}	5.48 ± 4.1^{a}	3.5 ± 1.8^{a}
*Hp*U6	28.78 ± 4.18^{ab}	27.15 ± 4.0^{bcd}	21.45 ± 3.6^{abc}	18.13 ± 1.3^{cd}	12.14 ± 2.4^{bc}
*Hp*G1	47.7 ± 4.5^{cd}	43.5 ± 4.7^{h}	36.26 ± 3.7^{d}	30.44 ± 1.6^{e}	20.1 ± 2.9^{d}
*Hp*G2	50.28 ± 4.2^{d}	45.88 ± 4.0^{h}	26.66 ± 4.6^{bcd}	15.7 ± 3.9^{abc}	5.48 ± 5.4^{ab}
*Hp*G3	53.44 ± 3.8^{d}	32.13 ± 3.9^{cdeg}	14.44 ± 2.14^{a}	9.90 ± 1.6^{ab}	3.78 ± 2^{a}
*Hp*G4	35.16 ± 4.13^{abc}	28.76 ± 3.28^{bcde}	18.90 ± 4.2^{ac}	13.12 ± 2.45^{abc}	4.13 ± 1.1^{a}
*Hp*G5	44.6 ± 5.51^{cd}	42.42 ± 4.5^{egh}	34.66 ± 3.9^{bd}	27.24 ± 4.4^{de}	18.00 ± 4.0^{acd}
*Hp*G6	48.84 ± 5.3^{cd}	33.18 ± 3.64^{cdeg}	29.88 ± 4.18^{cd}	22.14 ± 4.4^{ce}	15.70 ± 3.15^{cd}
*Hp*G7	51.51 ± 5.8^{cd}	40.28 ± 4.6^{egh}	27.80 ± 4.3^{cd}	12.90 ± 3.45^{abcd}	4.15 ± 2.1^{a}
*Hp*G8	47.62 ± 3.9^{ad}	35.66 ± 4.9^{degh}	29.22 ± 4.3^{cd}	15.78 ± 3.5^{abc}	4.6 ± 1.8^{ab}
Ref	51.24 ± 2.4^{d}	30.24 ± 1.5^{bcdeg}	15.4 ± 1.1^{ab}	10.8 ± 1.3^{abc}	1.34 ± 1.1^{a}

*Hp*U1 to *Hp*U6 are *H. pylori* strains isolated from ulcers, while *Hp*G1 to *Hp*G8 are *H. pylori* strains isolated from gastritis. Ref: *H. pylori* DSM21031 (reference strain). Similar superscript letters indicate no statistically significant differences (ANOVA with Tukey's HSD *post hoc* multiple range tests) among the various *H. pylori* strains for the same concentration of honey.

Table S2. Antibiotic susceptibility results from six antibiotics against *H. pylori* strains isolated from clinical samples (ulcers = 6, gastritis = 8) and a reference strain (*H. pylori* DSM21031).

Antibiotic	*HpU 1*	*HpU 2*	*HpU 3*	*HpU 4*	*HpU 5*	*HpU 6*	*HpG1*	*HpG2*	*HpG3*	*HpG4*	*HpG5*	*HpG 6*	*HpG 7*	*HpG8*	*Hp*Ref	*Clinical Breakpoints (mg/L)**	
																Susceptible	Resistant
Amoxicillin	R**	R	S	R	R	R	S	S	S	S	R	S	R	S	S	≤0.12	>0.12
Clarithromycin	R	S	R	S	R	R	R	S	S	S	S	S	S	S	S	≤0.25	>0.5
Metronidazole	S	R	R	S	S	R	S	S	R	R	S	S	R	R	S	≤8	>8
Tetracycline	R	S	S	R	R	S	S	S	S	R	R	R	R	S	S	≤1	>1
Levofloxacin	R	R	R	S	R	S	S	R	R	S	R	S	S	S	S	≤1	>1
Rifampicin***	R	S	R	R	S	R	S	S	R	S	R	S	S	S	S	≤1	>1

*Proposed by EUCAST (European Committee on AntimicrobialSusceptibility Testing) and the British Society for Antimicrobial Chemotherapy; **R:Resistant, S: Sensitive; ***Rifampicin was used to screen for rifabutin resistance since rifampicin E-tests are not available routinely (adapted from EUCAST).

Table S3. Diameter of the inhibition zones (mm, mean±St. Dev. produced from various solvent extracts (75% *v/v*) against 14 *H. pylori* strains isolated from ulcers (*n*=6) or gastritis (*n*=8) and a reference strain *H. pylori* DSM21031.

**Honey Extract (75% *v/v*)**	***Hp*U1***	***Hp*U2**	***Hp*U3**	***Hp*U4**	***Hp*U5**	***Hp*U6**	***Hp*G1**	***Hp*G2**	***Hp*G3**	***Hp*G4**	***Hp*G5**	***Hp*G6**	***Hp*G7**	***Hp*G8**	***H. pylori* DSM21031**
***n*-Hexane**	14.35 ± 3,5^{a}	12,85 ± 6,4^{a}	14,89 ± 5,3^{a}	16,41 ± 3,8^{a}	15,74 ± 3,8^{a}	16,81 ± 3,25^{a}	17,38 ± 3,8^{ab}	18,71 ± 4,5^{ab}	17,99 ± 3,7^{ab}	16,9 ± 3,5^{ab}	18,45 ± 4,3^{ab}	17,85 ± 3,6^{ab}	18,4 ± 3,8^{ab}	19,7 ± 4,2^{ab}	18,13 ± 3,9^{ab}
**Diethyl ether**	22,1 ± 5,7^{a}	19,5 ± 3,7^{a}	19,8 ± 4,7^{a}	19,88 ± 5,3^{a}	19,8 ± 2,9^{a}	19,87 ± 3,7^{ab}	23,7 ± 5,4^{ab}	25,1 ± 4,7^{b}	22,35 ± 4,7^{a,b}	25,8 ± 4,7^{bc}	26,4 ± 3,1^{b}	22,7 ± 2,8^{ab}	22,78 ± 3,8^{b}	22,74 ± 2,7^{b}	23,12 ± 3,8^{b}
**Chloroform**	14,7 ± 5,7^{a}	15,4 ± 2,8^{a}	14,18 ± 2,9^{a}	15,1 ± 3,9^{a}	15,78 ± 5,2^{a}	14,5 ± 2,8^{a}	12,1 ± 2,1^{a}	13,24 ± 3,4^{a}	12,7 ± 3,8^{a}	13,5 ± 3,8^{a}	12,8 ± 2,8^{b}	13,7 ± 3,8^{a}	12,75 ± 2,8^{a}	13,7 ± 2,8^{a}	13,9 ± 2,8^{a}
**Ethyl acetate**	25,42 ± 4,7^{a}	23,78 ± 4,7^{a}	24,58 ± 3,5^{a}	23,85 ± 4,8^{a}	25,45 ± 3,8^{a}	25,05 ± 2,7^{b}	24,78 ± 6,1^{b}	27,35 ± 2,1^{b}	25,78 ± 2,5^{b}	27,8 ± 1,8^{c}	25,78 ± 3,2^{b}	26,58 ± 3,5^{b}	26,85 ± 2,4^{b}	26,65 ± 2,5^{b}	26,35 ± 2,4^{b}

*Strains *Hp*U1–*Hp*U6 isolated from ulcers and *Hp*G1–*Hp*G8 from gastritis. Different superscript letters in columns indicate statistically significant differences (ANOVA with Tukey's HSD *post hoc* comparison, $p<0.05$) in the diameter of the inhibition zone between the solvent extracts within each *H. pylori* strain.

Table S4. MIC_{95} of solvent extracts (% *v*/*v*) of each honey against *H. pylori* strains isolated from ulcers and the reference strain DSM21031.

Solvent	N*	*Hp*U1	N*	*Hp*U2	N*	*Hp*U3	N*	*Hp*U4	N*	*Hp*U5	N*	*Hp*U6	N*	*Hp* DSM21031
n-hexane	32	10.23 ± 2.25^{4,c}	27	6.42 ± 4.16^{3,b}	7	7.78 ± 3.55^{3,bc}	11	5.97 ± 3.29^{3,ab}	6	5.83 ± 3.41^{2,3,ab}	9	6.38 ± 2.82^{3,ab}	21	4.28 ± 2.45^{3,a}
Diethyl ether	36	3.11 ± 2.84^{1,d}	39	0.93 ± 0.96^{1,ab}	41	2.13 ± 2.26^{1,c}	29	0.96 ± 0.65^{1,ab}	37	1.51 ± 1.58^{1,bc}	46	1.02 ± 0.64^{1,ab}	48	0.52 ± 0.76^{1,a}
Chlorofor m	25	7.3 ± 2.78^{3,b}	22	4.26 ± 2.36^{2,a}	5	5.0 ± 1.76^{2,3,ab}	12	6.83 ± 3.48^{3,b}	13	6.21 ± 3.68^{3,ab}	17	6.45 ± 4.07^{3,b}	20	9.85 ± 2.27^{4,c}
Ethyl acetate	28	4.82 ± 2.03^{2,ab}	38	2.96 ± 2.51^{2}	34	3.46 ± 3.44^{2,abc}	33	3.35 ± 3.27^{2,ab}	32	3.99 ± 3.24^{2,bc}	50	2.70 ± 2.80^{2,a}	36	2.29 ± 1.25^{2,a}

* Number of samples with inhibitory effect out of 50 oregano honey tested samples. Different superscript numbers indicate statistically significant differences in rows while superscript numbers indicate statistically significant differences in columns (ANOVA with Tukey's HSD *post hoc* comparisons at 95%).

Table S5. Urease Inhibitory effect of pure and diethyl ether extracts of oregano honey against two *H. pylori* strains isolated from ulcers (*Hp*U1, *Hp*U2) and the reference strain DSM21031.

*H. pylori*Strain	Oregano Honey	N*	Effective Concentration (mg/mL)		
			Mean ± SD	Range	Median
*Hp*U1	Pure	43	91.3 ± 55.8**	12.5–200.0	100.0
	Diethyl ether extract	50	22.0 ± 18.8	3.0–100.0	12.5
*Hp*U2	Pure	49	57.5 ± 40.5**	6.25–200.0	50.0
	Diethyl ether extract	50	5.93 ± 3.32	1.56-12.5	6.25
DSM21031	Pure	49	28.3 ± 18.3**	12.5–100.0	25.0
	Diethyl ether extract	50	4.43 ± 2.33	1.56–12.5	3.125

* Number of samples with inhibitory effect out of 50 oregano honey tested samples. **Statistically significant differences between pure and diethyl ether extract effective concentration according to the Mann–Whitney (Wilcoxon) W-test ($p<0.01$).

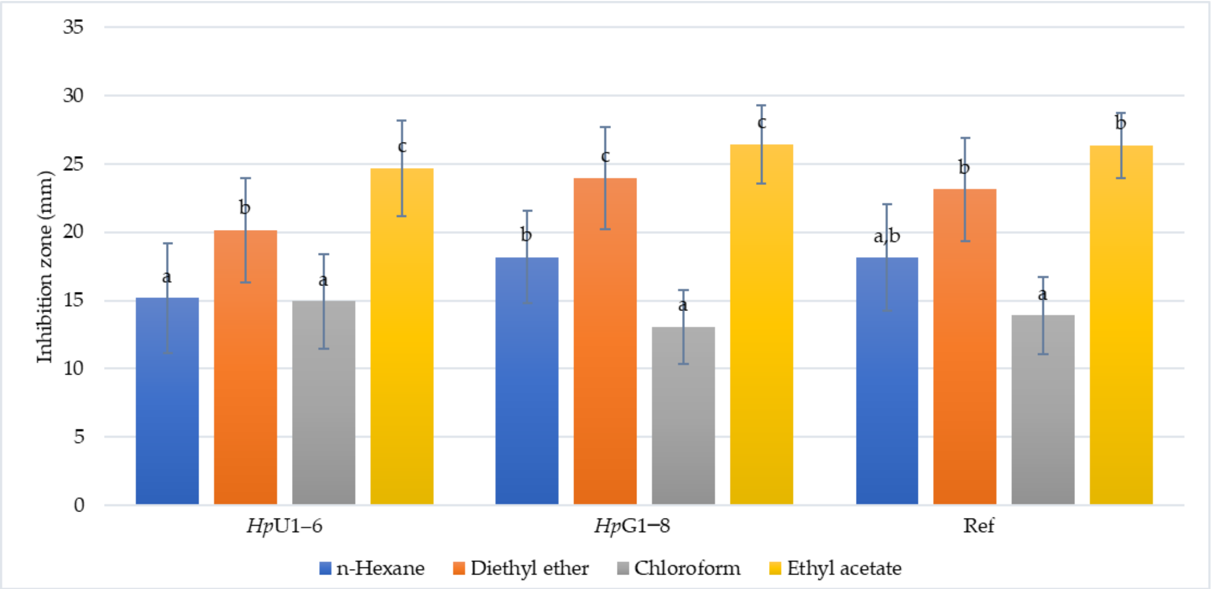

Figure S1. Mean diameter of inhibition zones from various solvent extracts against ulcer (*Hp*U1–6, n=6), gastritis (*Hp*G1–8, n=8), and the reference strain of *H. pylori* DSM21031. Different letters above bars indicate statistically significant differences (ANOVA with Tukey's HSD, $p<0.05$) for each group of strains.

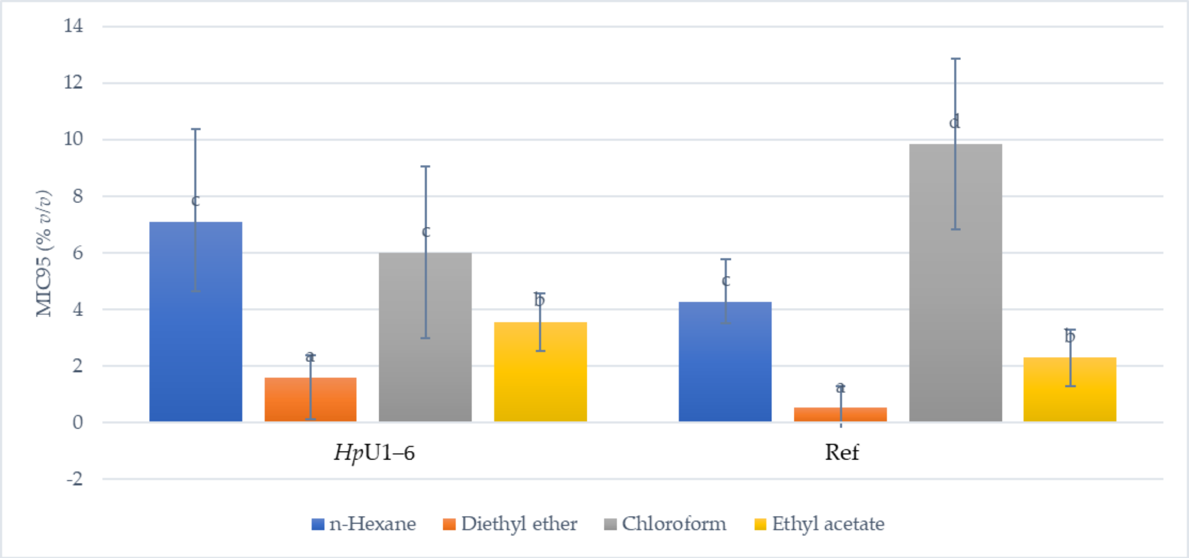

Figure S2. MIC95 values (% *v*/*v*) of *h*-hexane, diethyl ether, chloroform, and ethyl acetate oregano honey extracts against (6) *H. pylori* clinical strains (*Hp*U1–*Hp*U6) isolated from ulcers and a reference strain (*H. pylori* DSM210321).Different letters above bars indicate statistically significant differences (ANOVA with Tukey's HSD, $p<0.05$) for each group of strains.
